# Promoting improved utilization of laboratory testing through changes in an electronic medical record: experience at an academic medical center

**DOI:** 10.1186/s12911-015-0137-7

**Published:** 2015-02-22

**Authors:** Matthew D Krasowski, Deborah Chudzik, Anna Dolezal, Bryan Steussy, Michael P Gailey, Benjamin Koch, Sara B Kilborn, Benjamin W Darbro, Carolyn D Rysgaard, Julia A Klesney-Tait

**Affiliations:** Department of Pathology, University of Iowa Hospitals and Clinics, Iowa City, IA 52242 USA; Department of Anesthesia, University of Iowa Hospitals and Clinics, Iowa City, IA 52242 USA; Stead Family Department of Pediatrics, University of Iowa Hospitals and Clinics, Iowa City, IA 52242 USA; Department of Internal Medicine, University of Iowa Hospitals and Clinics, Iowa City, IA 52242 USA

**Keywords:** Blood cell count, Electronic health records, Medical informatics, Serum albumin, Vitamin D

## Abstract

This case study over time describes five years of experience with interventions to improve laboratory test utilization at an academic medical center. The high-frequency laboratory tests showing the biggest declines in order volume post intervention were serum albumin (36%) and erythrocyte sedimentation rate (17%). Introduction of restrictions for 170 high-cost send-out tests resulted in a 23% decline in order volume. Targeted interventions reduced mis-orders involving several “look-alike” tests: 1,25-dihydroxyvitamin D, 25-hydroxyvitamin D; manganese, magnesium; beta-2-glycoprotein, beta-2-microglobulin. Lastly, targeted alerts reduced duplicate orders of germline genetic testing and orders of hepatitis B surface antigen within 2 weeks of hepatitis B vaccination.

**Electronic supplementary material:**

The online version of this article (doi:10.1186/s12911-015-0137-7) contains supplementary material, which is available to authorized users.

## Introduction

Laboratory testing is an integral part of modern medicine, with test results influencing diagnosis, prognosis, and management of disease [1,2]. With increasing concerns about the overall rising costs of healthcare, a number of studies have analyzed utilization of laboratory testing. Inappropriate laboratory utilization (‘misutilization’) may include underutilization (not ordering clinically indicated testing) and overutilization (ordering tests too often), as well as ordering incorrect testing [3-15]. Laboratory testing contributes significantly to the overall cost of hospitalization. Moreover, misutilization of laboratory testing can potentially result in substantial downstream effects including iatrogenic blood loss, patient angst, invasive procedures, follow-up testing, and unnecessary specialist referrals [3,8,16-20]. “Defensive” medicine practice is one factor underlying misutilization that can contribute substantial downstream costs including excess hospitalization [21].

Over the last several decades, a number of studies have described efforts to improve laboratory utilization [4,7,22-26]. The interventions depend on the particular type of testing being targeted. Routine, automated laboratory tests such as electrolyte and complete blood count (CBC) panels have been identified in multiple studies as being prone to overutilization, particularly in the inpatient setting where such testing may be ordered daily or even more often without strong clinical indication [22,27]. Interventions to tackle overutilization of automated tests include limits on repetitive ordering, posting pricing of laboratory testing in the order entry system, and providing information to ordering providers on their ordering patterns relative to other providers [12,28-31]. To normalize data and reduce variability across patient populations, provider ordering patterns may be compared within specific diagnosis classifications (e.g., using diagnosis-related groups, DRGs, in the United States) or with patient data normalized to a variety of available severity index classifications [8,17].

Another category of laboratory testing that is frequently targeted in an effort to improve laboratory utilization are low-volume but high cost tests such as panels for genetic testing or autoantibodies [3,20,32,33]. In some cases, these panels may have direct costs of thousands of dollars (sometimes paid directly to external reference laboratories by hospitals, clinics, or laboratories) yet have poor reimbursement by payers. Some reference laboratory panels also include reflex algorithms that may generate substantial costs beyond the base charges. Inappropriate utilization of high cost, high complexity testing can have undesirable downstream effects that include unneeded additional testing, invasive procedures, and patient distress for borderline abnormal results. Analogous to pharmacies, some institutions have developed “laboratory test formularies” that place tiered restrictions on ordering of certain tests [34,35]. Restrictions for specific testing can include need for pre-approval by pathology or another designated group prior to ordering or limitation of ordering of certain tests to specific specialties (e.g., esoteric coagulation tests by hematology/oncology specialists).

The widespread use of electronic medical records (EMRs) and computerized provider order entry (CPOE) systems has an impact on laboratory test utilization. Providers working with computerized systems can potentially access thousands of possible laboratory tests from online test menus [23,24,29,36-38]. Available options for test order frequency and educational prompts may influence ordering patterns. Moreover, similarly named tests may be confused, resulting in misutilization.

We present a case study over time of EMR-based strategies to optimize laboratory test utilization efforts at a 711 bed academic medical center in the United States. These included efforts targeted at high-cost reference laboratory tests, duplicate genetic tests, high-volume automated tests, and look-alike tests. These data add to the cumulative literature on methods to promote better utilization of clinical laboratory testing.

## Methods

The data described in this manuscript involved multiple projects with University of Iowa Institutional Review Board (IRB) approval. These included IRB identification numbers 201311748 and 201407791(improving lab test utilization), 201404755 and 201208751 (drug of abuse testing), 201210797 (automation in clinical laboratory), and 201210796 (vitamin D testing). Some of the initiatives originated with quality improvement/management projects by pathology residents and fellows. The University of Iowa Hospitals and Clinics (UIHC) is a 711 bed state academic medical center that serves as a tertiary/quaternary care center. The medical center includes intensive care units (neonatal, medical, pediatric, surgical/neurologic), an emergency treatment center, and outpatient clinics. The electronic medical record (EMR) for the UIHC system changed to Epic (Epic Systems Inc., Madison, WI, USA) in May 2009, with uploading of historical data from paper charts and the previous EMRs back to 1996. All interventions described in this study occurred after the change in EMR in May 2009. The retrospective analysis covers the time period from May 2, 2009 until July 22, 2014.

### Organization of information systems and pathology testing

Throughout the entire period of retrospective study, computerized provider order entry (CPOE) within the UIHC system (including hospital units and outpatient clinics) was available in Epic to licensed independent providers. Add-on orders can also be placed within Epic. The laboratory information system (LIS) was Cerner (Kansas City, MO, USA) “Classic”, version 015, for all pathology testing. The EMR and LIS are managed by University of Iowa Hospitals Computing and Information Services. For provider orders sent on paper or transmitted verbally (e.g., by phone), laboratory-initiated orders were placed by clinical laboratory staff in the Cerner LIS. The UIHC core laboratory uses Data Innovations (South Burlington, VT) Instrument Manager (“Middleware”) for interfacing of instruments and for control of reflexive rules for automated tests [39]. Duplicate cancellation of testing was done within the Cerner LIS.

Pathology services at UIHC are primarily provided by clinical laboratories within the Department of Pathology (core chemistry/hematology laboratory, microbiology/molecular laboratories, DeGowin Blood Center, two critical care laboratories, and anatomic pathology laboratories). Send-out testing to reference laboratories is managed by the Department of Pathology. A hospital subcommittee (the Diagnostics Services Subcommittee) provides oversight of laboratory and radiology testing. This subcommittee reports to the Hospital Advisory Committee and includes physicians and administrators from the Department of Pathology, Department of Radiology, and multiple clinical departments (e.g., Department of Medicine, Department of Pediatrics, and Department of Surgery). In the time period of retrospective study, the Diagnostic Services Subcommittee and Hospital Advisory Committee supported initiatives to improve utilization of laboratory testing. This included formation of working groups to address specific issues such as management of very high-cost genetic laboratory testing.

### Original settings of the EMR

The EMR for UIHC changed in May 2009. Table 1 summarizes the settings of the EMR at go-live with respect to laboratory testing. For CPOE ordering of laboratory tests, the default setting allowed for a large range of possible frequencies in repetitive ordering of the test from a single order. Order sets created in the new EMR included conversion of prior order sets from the prior EMR and also paper requisitions. Checks to prevent duplicate ordering of tests were limited to cancellation of the exact same test ordered on the same accession number. Importantly, the original settings did not provide cancellation in cases where an individual test was duplicative of the same test as part of a panel (e.g., plasma sodium ordered on same accession number as the basic metabolic panel, which also included a plasma sodium). There were few prompts specific to tests. All send out testing had a generic warning stating that such testing should have approval of the attending physician of record. Pricing of laboratory testing was not available at EMR go-live; this was a change from the prior EMR, which included the Medicare charges for most laboratory testing.

**Table 1:** **Original settings in electronic medical record**

**Variable**	**Setting at electronic medical record (EMR) go-live May 2009**
Allowable frequencies for repetitive ordering of laboratory tests	All frequencies possible (ranging from multiple times daily to once per week)
Review of electronic order sets	Limited review by pathology; many order sets converted without major modification from paper requisitions or from previous EMR
Duplicate ordering of tests	Limited to cancellations by laboratory information system if same test(s) ordered on exact same collect time
Send-out tests	Generic prompt on all send-outs (i.e. not customized to any particular testing)
Restriction of tests requiring approval (‘laboratory formulary’)	No
Laboratory test charges	Not posted in EMR

### Statistical and cost analyses

Statistical analysis was done in SPSS (PASW Statistics 18, Chicago, IL, USA). Non-parametric Mann–Whitney tests were used for comparison of utilization between pre- and post-intervention periods. Data were normalized to number of patient days.

Cost savings of interventions within the EMR were estimated based on direct costs of testing, using decreases in testing post-intervention compared to the previous year pre-implementation. The cost analysis of in-house testing included labor (e.g., bench technologists, laboratory assistants) and reagents/supplies (calibrators, controls, pipettes, dilution tubes, etc.). The direct costs of reference laboratory testing included labor and reagents/supplies to process specimen for send-out and the invoice costs paid by UIHC to the reference laboratory.

## Results

### Interventions put in place

Over the 3 years following installation of the new EMR in 2009, interventions were put in place to promote better, more cost-effective utilization of laboratory testing. The broad changes are summarized in Tables 2, 3, 4 and 5 and included: limitations on selectable frequency of ordering of laboratory testing, expanded duplicate checking, removal of the generic send out testing warning, introduction of a variety of targeted test warnings based on data analysis, posting of laboratory test charges in CPOE order entry, and implementation of a send out test formulary. Additional file 1 details the maximum allowable frequency for specific tests that could be placed in a single order (Additional file 1A) and also details restrictions on selected send-outs (Additional file 1B). Additional file 2 contains the specific verbiage found in warning prompts and best practice alerts. These interventions occurred at various time points; in some cases, multiple interventions affected a single test. The following sections summarize the effects of these interventions.

**Table 2:** **Changes to electronic medical record affecting laboratory test ordering**

**Variable**	**Intervention**	**Date(s) of intervention**
Allowable frequencies for repetitive ordering of laboratory tests	Customized to tests	July 2012
Review of electronic order sets	All order sets with laboratory tests reviewed by pathology (pathology informatics and medical director) prior to release to production environment	September 2009
Duplicate ordering of tests	• Expanded duplicate checking to catch overlap of testing (e.g., glucose ordered individually at same time as basic metabolic panel that also included glucose order)	• October 2009
	• Instituted Best Practice Alert warning for attempt at duplicate orders of germline genetic tests	• January 2011
Send-out test warnings	• Removed generic send-out test prompt	May to July 2012
	• Instituted specific warnings with hard-stops customized to tests to include one or more of the following:	
	- High cost and very high cost warnings	
	- Long turnaround time warnings	
	- Genetic counseling prompt	
	- Reflex testing warning (if applicable)	
Restriction of tests requiring approval (formulary)	Instituted restrictions on 170 tests that required pathologist or clinical specialist approval prior to ordering	July 2012
	- 164 tests required pathologist approval	
	- 4 tests required infectious disease attending approval	
	- 2 tests required neurology attending approval	
Interventions targeting specific tests	Instituted based on review (see Table 3)	Various times 2009-2012
Laboratory test charges	Posted in EMR for most tests (either as discrete charges or range of possible charges)	July 2012

**Table 3:** **Examples of specific interventions within electronic medical record order entry system**

**Test(s)**	**Problem**	**Intervention**	**Date of intervention**
1,25-Dihydroxyvitamin D	Mis-orders (25-hydroxyvitamin D intended)	• Specific warning prompt	October 2009
		• Education	
		• Correction of order sets	
Manganese	Mis-orders (usually erroneously ordered for magnesium)	• Specific warning prompt	November 2009
Hepatitis B surface antigen	False positives due to recent administration by hepatitis B vaccine	Best Practice Alert if testing ordered within two weeks of recent vaccination	September 2011
Beta-2-microglobulin	Mis-orders for beta-2-glycoprotein, particularly in obstetrics	• Specific warning prompt	June 2010
		• Correction of order sets	
Complete blood count (CBC) with differential	Repetitive over-ordering of CBC with differential when CBC alone would suffice	Restricted repetitive ordering options of differential to once daily	June 2012

**Table 4:** **Maximum repetitive order frequencies available by single order**

**Maximum repetitive frequency allowed in a single order**	**Examples of tests in this category**
Once	CBC differential
	Blood smear morphology review by technologist or pathologist
	Albumin
	Alpha-fetoprotein
	Amylase
	Angiotensin converting enzyme
	C-Peptide
	C-Reactive Protein
	CA-125
	Epstein-Barr virus serologies
	Hemoglobin A1C
	Hemoglobin electrophoresis
	Lipase
	Lipids (cholesterol, high-density lipoprotein, low-density lipoprotein, triglycerides)
	Molecular pathology (e.g., Factor V Leiden genotyping)
	Phosphorus
	(Most) Send-out testing
	Thyroid stimulating hormone
Every 72 hours	Erythrocyte Sedimentation Rate
	Serum free light chains (kappa/lambda)
Daily	Basic metabolic panel
	Electrolyte panel
Once per week	*Aspergillus* galactomannan
	Histoplasma antigen
	Serum and urine protein electrophoresis

**Table 5:** **Most common issues detected by and subsequently corrected following pathology review of draft EMR order sets**

**Issue**	**Most common examples that were corrected**
Erroneous inclusion of test that had similar name to intended test	• Lactate dehydrogenase (LDH) isoforms instead of LDH
	• Amylase isoforms instead of amylase
	• Confusion of beta-2-microglobulin and beta-2-glycoprotein
Confirmatory/specific test used instead of common screening test	• Hepatitis B DNA instead of hepatitis B surface antigen
	• Hepatitis C RNA instead of hepatitis C antibody
	• HIV viral load instead of HIV antigen/antibody combo assay
Repetitive ordering of common tests, either too frequent time intervals or for too long duration or both	• Basic metabolic panel (sodium, potassium, carbon dioxide, chloride, calcium, blood urea nitrogen, creatinine, glucose)
	• Complete blood count (with or without differential)
	• Blood gases
	• Lactate
	• Liver enzymes (e.g., alanine aminotransferase, aspartate aminotransferase)
	• Osmolality
Selection of obsolete testing or low yield testing	• *Chlamydia* culture instead of *Chlamydia* PCR
	• Antibody assays for fungi endemic to Iowa (e.g., *Histoplasma*, *Blastomyces*) instead of higher yield antigen or culture-based tests

### Restrictions on send out testing

**Figure 1:**
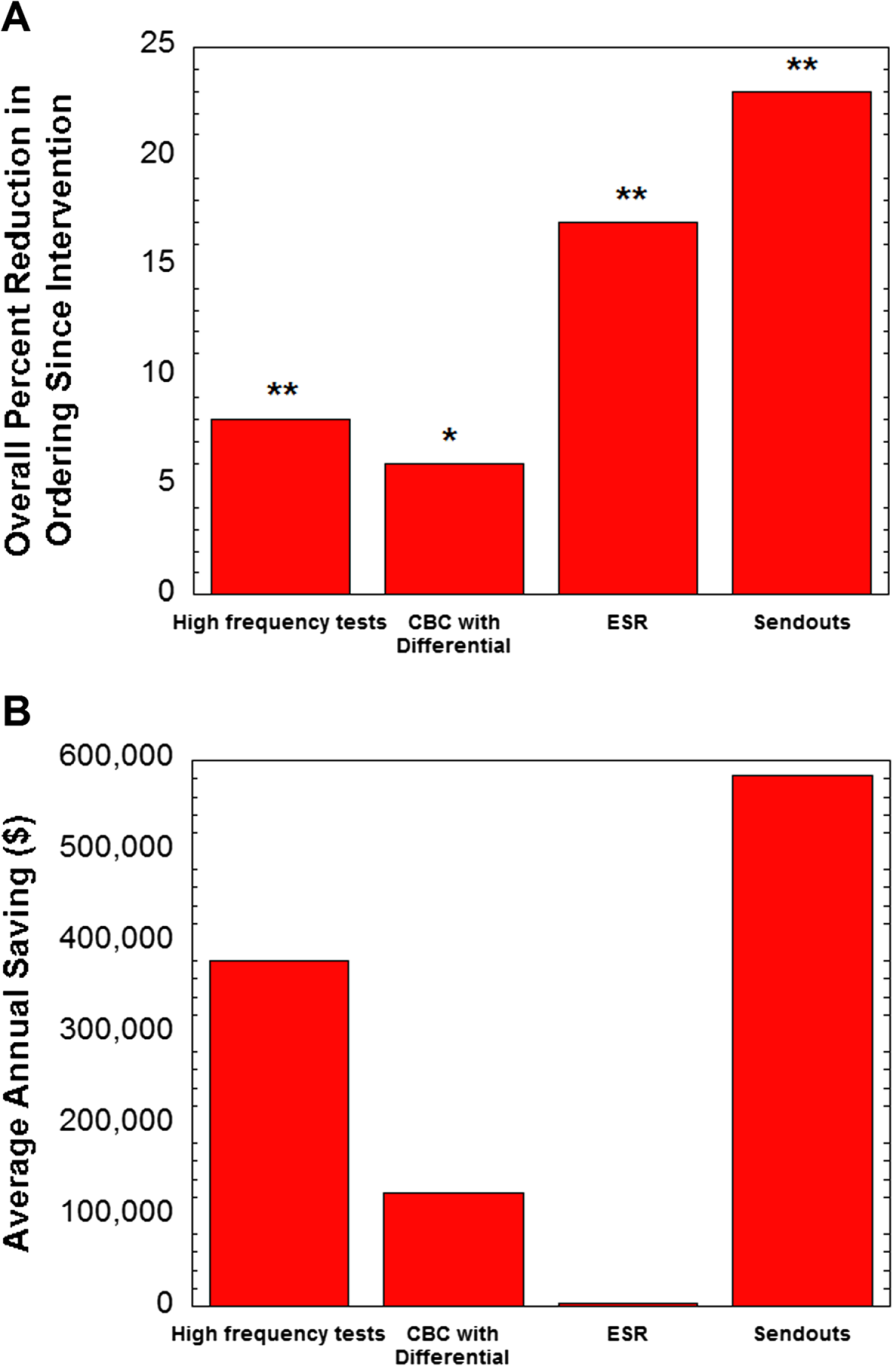
**Changes in laboratory test ordering.** Data is broken down into high frequency tests (core laboratory chemistry and hematology testing excluding CBC with differential), CBC with differential, erythrocyte sedimentation rate (ESR), and restricted send-out testing with effects on **(A)** frequency of ordering (adjusted for patient days) and **(B)** average annual savings. The pre-intervention period was two years leading up to interventions (changes in ordering frequency options; institution of restricted send-out testing) in July 2012. The post-intervention period is two years following that into July 2014. *P < 0.005, **P < 0.001.

Beginning in July 2012, a total of 170 send out tests became restricted (Table 2, Additional file 1B), requiring approval from a pathologist (164 tests), an infectious disease attending (4 tests), or a neurology attending (2 tests). Post implementation, orders for restricted send outs decreased overall by 23% with an overall annual institutional savings in direct costs of approximately $600,000 (Figure 1). The tests with the largest reduction in ordering were (summarized as average tests/month pre-intervention to tests/month post-intervention): serum paraneoplastic autoantibody panel (8.0 to 2.3 tests/month), vitamin B_12_ panel (as opposed to a single vitamin B_12_ level; 8.7 to 2.1 tests/month), cerebrospinal fluid paraneoplastic autoantibody panel (3.8 to 1.7 tests/month), and Fibrospect II® (3.8 to 1.8 tests/month). These four tests required pathologist approval. Analysis of the calls received by pathology residents and faculty related to approval of restricted send out tests over a twelve month period showed that 41% of the calls were seeking approval for the paraneoplastic autoantibody panels. Overall, 60% of requests for approval of restricted send out tests were not granted. Our analysis does not capture providers who started to order the test but did not attempt to seek approval after seeing warnings and/or the need for approval.

Of the four HIV tests requiring infectious disease attending approval (HIV Genotyping, HIV-1 Proviral DNA, HIV-2 Proviral DNA, HIV Phenotyping), overall orders rose by an average of 0.3 tests per month, which appeared to be driven by an increase in HIV genotyping orders, mainly directly ordered from Infectious Disease outpatient clinics. An analysis of ordering patterns showed these tests were ordered appropriately and reflected an increase in the HIV-positive population treated at the medical center. Other than HIV Genotyping, orders for the other 3 restricted HIV tests all dropped post intervention, and none showed evidence of misutilization. The two tests that required neurology attending approval, MUSK Antibody Panel and Myasthenia Gravis Reflexive Panel, decreased by 0.8 and 0.4 tests/month, respectively.

### Impacts on high-volume testing

The major interventions that targeted high volume laboratory tests were introduction of electronic duplicate test cancellation and modification of the allowable options for repetitive frequency that could be selected in a single electronic order (Table 4, Additional file 1). Prior to the introduction of automated electronic duplicate test cancellation in October 2009, a warning prompt fired to warn the ordering provider of duplicate ordering and provided option for test cancellation. Data following EMR go-live in May to September 2009 showed that this warning prompt system was ineffective and overridden more than 95% of the time. The intervention on selectable test frequencies in July 2012 did not prevent providers from placing multiple separate orders at different points in time to accomplish the same frequency.

Overall, the order volume of high-frequency tests (adjusted for patient days) declined by 8% following the changes in test order frequency in the EMR ordering menus (Figure 1). The laboratory tests that showed the biggest changes in order volume post intervention were serum albumin (36% decline) and erythrocyte sedimentation rate (ESR) (17% decline). Orders of a differential with a CBC declined 6%. The overall savings in direct costs was estimated at slightly less than $400,000 annually (Figure 1; see Methods for details). The decrease in albumin ordering was consistent with data prior to intervention showing that 19.6% of albumin orders were repeats within one day of a prior result and 29.9% were repeats within one week of prior order in a nine month period spanning 2009–2010. The decrease in ESR ordering was also consistent with pre-intervention data showing that 25.1% of ESR orders were repeats within 48 hours of a previous order in a six month period in 2009.

### Duplicate genetic tests

In January 2011, a Best Practice Alert (BPA) was put into effect to catch attempts at ordering a germline genetic test that had been previously ordered and resulted in the EMR. This BPA did not apply to genetic testing for somatic mutations (such as in cancer testing) and gave the ordering provider a warning that the test had already been performed and gave the option to cancel the testing or to provide a reason why the testing should be re-performed. The duplicate genetic testing BPA was effective in preventing 99 orders, 59 of which were attempted orders for a combined panel of Factor V Leiden/prothrombin mutations and 20 were for hemochromatosis mutation analysis. Additional duplicate genetic tests prevented by the BPA included FMR1 (fragile X mutation analysis, n = 9), cystic fibrosis mutation analysis (n = 4), and SMN1 (spinal muscular atrophy,n = 2), along with five additional tests that were prevented one time each (CYP21A2, FBN1, MEN1, RET, SCN1A gene sequencing panels). Of note, eight of the duplicate orders prevented would have exceeded $1,000 each in direct reference laboratory costs to the institution. Seven duplicate genetic test BPAs were overridden. Of these cases, five were due to the testing having changed (e.g., more extensive mutation analysis now available or a significant change in test analytical methodology) since the original testing performed.

### Hepatitis B surface antigen orders

In September 2011, a BPA was put in place to prevent ordering of laboratory testing for hepatitis B surface antigen within two weeks of administration of a dose of hepatitis B vaccine (either individual formulations of the vaccine or formulations combined with hepatitis A vaccine). The basis for this BPA was published in a retrospective study showing that recent administration of hepatitis B vaccine was a frequent cause of weakly positive hepatitis B surface antigen results at UIHC, particularly in the adult dialysis population [40]. The findings at UIHC were similar to previous published reports on this phenomenon [41-46]. Since the September 2011 go-live of the hepatitis B BPA, 68 orders were prevented. This dropped the overall rate of laboratory testing of hepatitis B surface antigen within two weeks of a vaccine dose from 2.0/month (before intervention) to 0.3/month (post intervention). Of the 68 orders that were prevented, 23 were on inpatient units, 19 were in solid organ transplant clinic, 10 were in primary care clinic, and five were in dialysis clinic. Six of the patients were infants age 13 months or younger at time of order, and there were an additional five pediatric patients between the ages of two years and 14 years at the time of ordering.

### “Look-alike” laboratory tests

Two quality improvement projects within the Department of Pathology focused on mis-orders of laboratory tests with similar looking names. These analyses revealed two pairs of tests which were getting frequently confused: magnesium and manganese; beta-2-glycoprotein and beta-2-microglobulin. Analysis of manganese orders estimated that 9.2% of orders were errors in which magnesium was the intended order. This was evident in chart review where no evidence could be found of why manganese was ordered. At UIHC, the most frequent intended orders for manganese were either related to nutrition (most often issues with parenteral nutrition, in which manganese overload can occur in specific populations such as in premature infants [47,48]) or specific workup of possible manganese toxicity, such as may occur in individuals whose occupation can involve manganese exposure (e.g., welding) [47]. To prevent mis-orders of manganese, a warning prompt was put in place in November 2009. This intervention was highly effective with only a single mis-order identified post intervention.

For beta-2-glycoprotein and beta-2-microglobulin, possible mis-orders were noted by clusters of orders for beta-2-microglobulin for obstetric patients in 2009 and 2010. The main clinical utility for serum beta-2-microglobulin is in the workup of multiple myeloma [49] and this would be an unusual order in the obstetric population. In a period of 10 months, at least 11 mis-orders for beta-2-microglobulin occurred in obstetric patients. In all 11 patients, the workup at the time of the outpatient encounter was for hypercoagulable disorder (including antiphospholipid antibody syndrome) as a possible explanation of recurrent pregnancy loss, with the intended order being beta-2-glycoprotein, a factor known to be associated with recurrent pregnancy loss in the setting of antiphospholipid antibody syndrome [50]. Identification of this problem led to the discovery that an order set for recurrent pregnancy loss used by Obstetrics/Gynecology erroneously had beta-2-microglobulin instead of beta-2-glycoprotein. Correction of the order set, provider education, and a warning prompt for beta-2-microglobulin orders was highly effective in preventing future mis-orders. Since the introduction of the warning prompt in June 2010, only 3 mis-orders for beta-2-microglobulin have been noted for obstetric patients. These were sporadic mis-orders by 3 different providers and not temporally associated with one another or with use of an order set.

### Vitamin D mis-ordering

**Figure 2:**
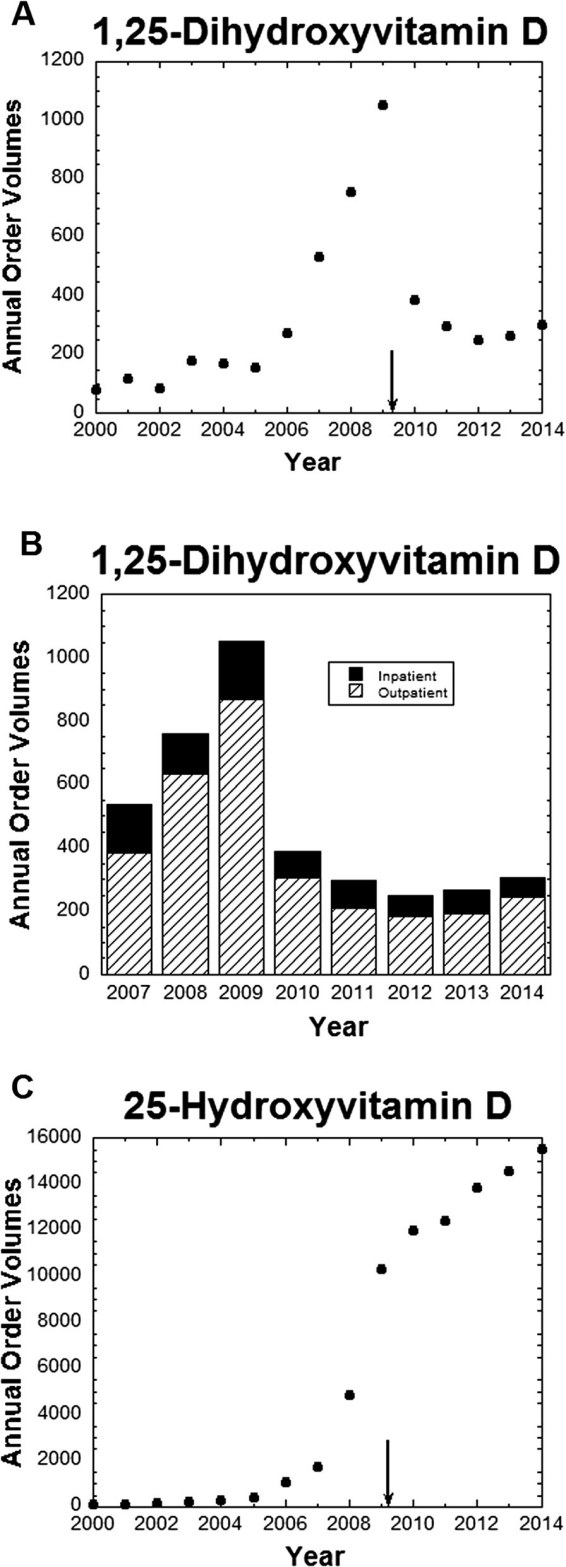
**Ordering patterns of 1,25-dihydroxyvitamin D and 25-hydroxyvitamin D. (A)** Annual test volumes for 1,25-dihydroxyvitamin D from 2000 to 2014 (annual volume for 2014 estimated based on data through 7/22/2014); **(B)** Annual test volumes for 1,25-dihydroxyvitamin D from 2007 to 2014 (annual volume for 2014 estimated based on data through 7/22/2014), broken down by location of order – inpatient unit or outpatient clinics; **(C)** Annual test volumes for 25-dihydroxyvitamin D from 2000 to 2014 (annual volume for 2014 estimated based on data through 7/22/2014). Subset of data for 25-dihydroxyvitamin D up to October 2012 has been previously published [53]. The arrows indicate date that new EMR was introduced.

An example of mis-ordering with much higher magnitude occurred with vitamin D testing. Following the switch to the new EMR in 2009, ordering of 1,25-dihydroxyvitamin D (calcitriol) increased substantially. Total annual orders for this send-out test were higher in 2006–2008 compared to 2000–2005 and reached their highest levels in 2009 (Figure 2A). 1,25-Dihydroxyvitamin D is the more biologically active form of vitamin D, but is not the appropriate test for routine nutritional screening of vitamin D status, for which 25-hydroxyvitamin D is preferred [51,52]. In fact, 1,25-dihydroxyvitamin D can change throughout the day for a patient and may provide a misleading measure of overall vitamin D status.

Analysis revealed that 43 EMR order sets had been constructed in 2009 that only contained 1,25-dihydroxyvitamin D and not 25-hydroxyvitamin D in the list of laboratory tests. In 10 cases, the order for 1,25-dihydroxyvitamin D was pre-selected; in 3 cases, the order was pre-selected and could not be unselected if the order set were utilized. Discussion with the providers assigned “ownership” of these 43 order sets revealed that the intent in every case was routine screening of vitamin D status, and thus the 1,25-dihydroxyvitamin D was not appropriate. All 43 order sets were corrected and a specific warning prompt was put in place for 1,25-dihydroxyvitamin D. Further interventions included a system-wide broadcast and targeted education efforts by pathology and endocrinology. These efforts were highly effective in limiting 1,25-dihydroxyvitamin D orders, which returned to ordering volumes similar to those in 2006 (Figure 2A). Breakdown of the 1,25-dihydroxyvitamin D orders by location of order showed that increased ordering of 1,25-dihydroxyvitamin D in 2007–2009 occurred in both outpatient clinics and inpatient units (Figure 2B). In contrast to 1,25-dihydroxyvitamin D, orders for 25-hydroxyvitamin D have continued to increase through 2014 (Figure 2C), as we have noted in a previous study [53].

## Discussion

A number of published studies have described efforts to improve laboratory test utilization [4,7,22-26]. Routine, automated tests such as electrolyte panels have been consistently identified as being overutilized, especially on inpatient units [22,27]. Moreover, this repetitive testing can lead to significant iatrogenic blood loss [16,19]. Our study found that the menu of choices for repetitive ordering of testing has a direct impact on ordering patterns. In particular, the largest impact was on albumin and ESR, two tests that change slowly (over the course of days and not hours) and thus should rarely need to be monitored daily or more often. The “default” configuration for the EMR at our institution was to have a wide range of frequency choices available for every laboratory test. For many tests, repetitive testing has no clinical justification and should not be available as an option.

Our case study over time adds to the published literature that active utilization efforts can impact low-volume, high-cost reference laboratory testing [3,20,32,33]. Some of these efforts are similar to the formularies used by pharmacy to manage medication usage [34,35]. In terms of decreased ordering, the biggest impact in our institution on restricted send-out testing was on the paraneoplastic autoantibody panels (serum and CSF) and an expanded vitamin B_12_ panel.

The issue of “look-alike” medications is an issue that has been actively targeted by pharmacies because of the potential for patient harm [54,55]. Our study uncovered several pairs of look-alike laboratory tests that were confused: 1,25-dihydroxyvitamin D and 25-hydroxyvitamin D; manganese and magnesium; beta-2-glycoprotein and beta-2-microglobulin. Of these, vitamin D mis-ordering was of the greatest magnitude and caused in part by dozens of electronic order sets that erroneously had 1,25-dihydroxyvitamin D instead of 25-hydroxyvitamin D in the list of laboratory tests. These results illustrate the importance of careful review of order sets prior to release to the production environment of the EMR. Poorly constructed order sets have the potential to magnify errors and even consolidate confusion (“if it is in an order set, it must be correct”).

Lastly, we found that targeted BPAs were successful in reducing duplicate ordering of germline genetic testing and preventing orders of hepatitis B surface antigen within 2 weeks of hepatitis B vaccination. Germline genetic tests should rarely need to be repeated unless there is concern of inaccurate test results (in which case the laboratory should be contacted to investigate) or the methodology and/or scope of testing have changed in a manner that warrants retesting in specific patients. The duplicate genetic test BPA most often prevented repeat testing of factor V Leiden/prothrombin and hemochromatosis mutation analyses.

The main limitation of our case study is that the analysis is retrospective and that multiple interventions were done over time that could impact laboratory test orders. The interventions put in place were not done as part of a controlled research study but evolved over time to improve clinical care. There is also the possibility that other factors not measured impacted laboratory test order. Nevertheless, it is hoped that the results described here provide useful experience for other institutions attempting to manage laboratory test utilization.

## Conclusions

Relatively simple changes in CPOE within EMR can have a significant impact on laboratory test utilization. Our case study is consistent with previous studies showing that development of utilization management for high cost, specialized laboratory testing is an effective tool.

## Additional files

Additional file 1:
**Maximum Allowable Test Frequencies and Restricted Send-outs.** Spreadsheet with complete data on allowable test frequencies per single order and details on restrictions of selected send-out tests. Additional file 2:
**Details on Prompts in the Electronic Medical Record.** File contains the verbiage of prompts for specific purposes in the electronic medical record.

## Abbreviations

BPA: Best practice alert
CBC: Complete blood count
CPOE: Computerized provider order entry
DRG: Diagnosis-related group
EMR: Electronic medical record
ESR: Erythrocyte sedimentation rate
UIHC: University of Iowa Hospitals and Clinics
