# Supplementary material for: Promoting improved utilization of laboratory testing through changes in an electronic medical record: experience at an academic medical center
Source: BMC Med Inform Decis Mak. 2015 Feb 22;15:11. doi: 10.1186/s12911-015-0137-7 (PMC4344785; doi:10.1186/s12911-015-0137-7)
Supplement: Additional file 2: — Details on Prompts in the Electronic Medical Record. File contains the verbiage of prompts for specific purposes in the electronic medical record. [file 12911_2015_137_MOESM2_ESM.docx]

**Additional File 2**

Details of Warning Prompts in the EMR

| **Prompt** | **Prompt Verbiage** |
| --- | --- |
| 1,25-Dihydroxyvitamin D | Measurement of Vitamin D (1,25-dihydroxy) is NOT useful in the routine screening for nutritional vitamin D deficiency. The preferred test is Vitamin D, 25-hydroxy. Is Vitamin D (1,25-dihydroxy) testing clinically indicated? |
| Beta-2-microglobulin | Verify that you want “Beta 2 microglobulin” and not “Beta 2 glycoprotein I IgG” or “Beta 2 glycoprotein I IgM”. Beta 2 glycoprotein is ordered mainly for hypercoagulable workup. |
| High-priced testing warning | [{Very}, {Extremely}] high-priced tests warning – charges from this test exceed $[{$250},{$500},{$750},{$1,000},{$2,000},{$5,000},{$10,000}]. I have made the patient aware of potential financial liability from this order. If orderd on inpatient encounter, I am aware that most reimbursement is by diagnosis related group (DRG) without specific coverage of diagnostic testing. |
| Long turnaround time warning | Extended turnaround time warning – expected turnaround time for this test is > [{1 week},{2 weeks},{4 weeks},{2 months}]. Ordering on inpatient encounters is strongly discouraged unless the test result is expected to impact care on the current inpatient stay. I certify I am aware of the long turnaround time for the smell test in that the order is clinically indicated. |
| Manganese | Verify that you want “Manganese” and not “Magnesium”. |
| Paraneoplastic Autoantibody Panel, Serum | The paraneoplastic autoantibody evaluation panel is very broad and more targeted workup (with generally faster turnaround time) is possible with the following alternative tests: (a) NMDA Receptor Antibodies, Serum and/or NMDA Receptor Autoantibodies, CSF; (b) Voltage-gated calcium antibodies, (c) Voltage-gated potassium channel antibodies, (d) Acetylcholine receptor binding antibody.  The paraneoplastic autoantibody evaluation panel is intended for workup of patients with paraneoplastic neurologic symptoms AND presence or suspicion of malignancy (especially breast carcinoma, small cell lung cancer, ovarian carcinoma, lymphoma, or neuroblastoma). General screening for unexplained neurologic symptoms in the absence of reasonable suspicion for malignancy has low diagnostic yield and can lead to false positives. I certify that this panel of testing is clinically indicated for this patient. |
| Reflex test warning | Depending on results of initial testing, the reference laboratory performing this test can initiate further reflex testing that will involve additional charges for the patient. I understand this and certify that ordering clinically indicated. |
| Restricted send-out requiring infectious disease attending approval | This mailout test has restricted ordering and requires approval from Infectious Disease attending (see drop-down menu to right). If approval is granted, select name of the Infectious Disease attending from the drop-down menu. |
| Restricted send-out requiring neurology attending approval | This mailout test has restricted ordering and requires approval from Neurology attending (see drop-down menu to right). If approval is granted, select name of the Neurologist from the drop-down menu. |
| Restricted send-out requiring pathologist approval | This test has restricted ordering on inpatient encounters and requires pathologist approval. Page XXX for pathologist covering mailouts approval. |
